# Effects of nurse delivered thoracic ultrasound on management of adult intensive care unit patients: A prospective observational study

**DOI:** 10.1016/j.ijnsa.2023.100135

**Published:** 2023-05-29

**Authors:** Thomas Smits, Micah Heldeweg, Amy Morreale Tulleken, Brian Verlaan, Lonneke Floor, Alwin Eijsenga, Erik Lust, Harry Gelissen, Armand Girbes, Paul Elbers, Pieter Roel Tuinman

**Affiliations:** a Department of Intensive Care Medicine, Amsterdam University Medical Centers, Location VUmc, Amsterdam, the Netherlands; b Amsterdam Cardiovascular Sciences Research Institute, Amsterdam UMC, Location: VUmc. De Boelelaan 1118, Amsterdam, 1081 HZ, the Netherlands; c Amsterdam Leiden Intensive care Focused Echography (ALIFE), the Netherlands; d Nursing Sciences, program in Clinical Health Sciences, University Medical Center Utrecht, Utrecht, the Netherlands

**Keywords:** Critical care nursing, Ultrasonography, Thorax, Lung, Heart, Cardiac output, Therapeutics, Observational study

## Abstract

**Background:**

Thoracic ultrasound is a valuable tool that helps diagnose cardiopulmonary disorders and guide management in intensive care unit patients. Intensive care unit nurses were trained to perform thoracic ultrasound examinations, after which they were named ‘UltraNurses’ for clinical recognizability. UltraNurses demonstrated rapid learning trajectories, but the impact on clinical-decision making remained unknown. The aim of this study was to investigate the effects of UltraNurse ultrasound on clinical management.

**Methods:**

This was a prospective observational single center study within a mixed medical and surgical intensive care unit. All adult patients with an indication for UltraNurse thoracic ultrasound were included. The study consisted of three steps: pre- and post- data collection, with the ultrasound examination conducted in-between these two steps. The examination consisted of a standardized ultrasound protocol aimed at the lungs and cardiac output. Primary outcome was what percentage of ultrasounds led to a change of management. Secondary outcomes included: percentage of changes executed within first 8 hours, frequency of pathology found, percentage of diagnosis change, and frequency of UltraNurse ultrasounds per shift.

**Results:**

A total of 102 ultrasound examinations were performed in 65 patients (89% mechanically-ventilated). Ultrasound examinations suggested changes of management in 26% of cases, of which 96% were executed within 8 hours. Most changes were within the nursing scope (56%), specifically: 44% of examinations changed fluid management. UltraNurse ultrasound detected pathology in 97% of cases. In 7% of cases, the diagnosis was changed, sometimes leading to life-saving interventions. UltraNurses performed one thoracic ultrasound examination per four shifts.

**Conclusion:**

In adult intensive care unit patients, UltraNurse thoracic ultrasound led to a change of management in more than a quarter of the cases, of which almost all were executed within the first 8 hours.

**Study registration:**

Netherlands Trial Registration NL9047, VUmc 2020.011 (prospectively registered on: 13–11–2020)

## 1 Background

Thoracic ultrasound is a valuable and increasingly-used bedside tool within daily intensive care unit (ICU)practice (Kendall et al., 2007; Lichtenstein et al., 2014). Thoracic ultrasound allows accurate and dynamic evaluation of cardiopulmonary pathologies within the ICU. Furthermore, thoracic ultrasound is bedside-available, repeatable, low cost, and relatively easy-to-learn and comes without harmful radiation (Lichtenstein et al., 2014; Haji et al., 2018; Heldeweg et al., 2022).

Thoracic ultrasound is normally performed by physicians in ICU patients and has been shown to have positive effects on clinical decision-making and management (Heldeweg et al., 2022; Xirouchaki et al., 2014; MLA Heldeweg et al., 2021). However, thoracic ultrasound performed by specialized nurses is not yet widespread, although it appears to have comparable accuracy (Mumoli et al., 2016). Moreover, a cohort of ICU nurses was recently trained in thoracic ultrasound. This training focused on widely-used and standardized ultrasound protocols aimed at the lungs through the Bedside Lung Ultrasound in Emergency protocol and cardiac output of the heart using the Velocity Time Integral (Lichtenstein, 2015; Lichtenstein and Mezière, 2008; Matta et al., 2019). These ICU nurses were seeking to obtain the ultrasound certification named ‘UltraNurse’. This term was chosen for clinical recognition and effective communication in high pressure situations. UltraNurses showed rapid learning trajectories in obtaining high-quality thoracic ultrasound examinations comparable to those of physicians and respiratory therapists (Morreale Tulleken et al., 2019; See et al., 2016).

Thoracic ultrasound performed by ICU nurses has the potential to improve clinical management, as ICU nurses are continuously at the bedside and are the first clinicians present when a patient develops cardiopulmonary deterioration. In such situations, a short interval to diagnosis and management is paramount. Moreover, daily management targets, such as the optimization of fluid balance, are in part handled by ICU nurses and could be guided by thoracic ultrasound (Heldeweg et al., 2021; Carpenito, 2012; Mccloskey Dochterman et al., 2007; Moorhead et al., 2018). Therefore, thoracic ultrasound may be a valuable extension of the ICU nursing scope of practice in supporting management of ICU patients.

However, the clinical implications of UltraNurse thoracic ultrasound examination remain uninvestigated (Morreale Tulleken et al., 2019). Also, there is a recent and broader call to gather more evidence on the effects of thoracic ultrasound on patient management (Bernstein and Wang, 2021).

Therefore, the primary objective of this study was to determine how frequently UltraNurse thoracic ultrasound led to a change of management. Secondary objectives were to determine what percentage of changes is executed within the first 8 hours, frequency of pathologic findings detected by ultrasound, frequency of clinical diagnosis change, and with what frequency an UltraNurse was able to perform an ultrasound examination. We hypothesized that UltraNurse thoracic ultrasound would lead to a change in management in about a third of examinations and that the suggested changes would be mostly executable within 8 hours.

## 2 Methods

### 2.1 Design and setting

This was a prospective, single center, observational study conducted at the mixed medical and surgical academic ICU of the Amsterdam University Medical Center—location VUmc, Amsterdam, The Netherlands. Patient recruitment took place between February and August of 2021.

### 2.2 Population

All adult patients admitted to the ICU who underwent a clinically-indicated UltraNurse thoracic ultrasound were included. As a result, multiple examinations for the same patient could be included. Patients were excluded when unable to undergo ultrasound examination; e.g., very agitated (Richmond Agitation-Sedation Scale ≥3) (Sessler et al., 2002). If there were technical particularities for which a patient was going to be excluded, these were to be noted on the case report form (see step 3 under study procedure).

### 2.3 Study procedure

#### 2.3.1 Step 1: Pre-ultrasound examination

Ultrasound examinations were integrated into conventional clinical practice on this ICU and were already regularly performed by clinicians; hence, only ultrasound examinations made by an UltraNurse during this period were included. When a patient presented a clinical indication for undergoing ultrasound, a certified UltraNurse would start the study procedure (see Supplement 1). If the clinical indications were acute, the ultrasound was performed immediately upon indication or request. All patients were eligible for undergoing ultrasound examinations, regardless of whether they were assigned to an UltraNurse. Clinical indications for undergoing an ultrasound examination were broad and initiated from the insights of the treating clinicians (ICU nurse or physician). Indications included cardiac or pulmonary worsening, as seen in deteriorating vital signs, blood gas panels, or physical examinations. Other indications were the monitoring of therapy or a specific diagnostic/management question regarding the ICU nursing scope of practice. Before UltraNurse thoracic ultrasound, the initial impression of clinical diagnosis and management was noted on the case report form (see Supplement 2).

#### 2.3.2 Step 2: Ultrasound examination

The UltraNurse thoracic ultrasound itself was performed with the SonoSite Edge II ultrasound device. The first part of the ultrasound examination focused on the lungs. The ventral lung ultrasound was performed with the linear probe in the ‘lung exam profile’; the lateral examination was performed with the phased-array probe in ‘cardiac exam profile’ (to filter artifacts). Both the brightness (2D) and motion (B-/M-)modes were used. The standardized Bedside Lung Ultrasound in Emergency-protocol was used and consisted of six-point thoracic assessment (see Supplement 3) (Morreale Tulleken et al., 2019). This protocol is able to distinguish signs indicative of the most common lung pathologies (Lichtenstein, 2015). The second part of the ultrasound examination focused on the cardiac output of the heart with the phased-array transducer through the standardized Velocity Time Integral from the apical window, obtained in the ‘cardiac exam profile’ (see Supplement 3) (Matta et al., 2019). For more clarification of which type of standardized training and certification the UltraNurses received, see Supplement 3 (Morreale Tulleken et al., 2019).

#### 2.3.3 Step 3: Post-ultrasound examination

After the ultrasound examination, the following data were registered on the case report form (see Supplement 2):

• Ultrasound findings (pathology, such as atelectasis, pulmonary edema, pleural effusion, etc.).
• Clinical contribution of the ultrasound (after UltraNurse-physician discussion): ‘no contribution’, ‘confirmation’, or ‘change of initial impression’.
• Change of prespecified initial diagnosis or management: if a change occurred, specification of type of diagnosis/management change.
• Any particularities possibly leading to exclusion (technical issues, poor echogenicity, drain interference, etc.).

To minimize bias, the clinical contribution was collectively scored by the physician and UltraNurse after the UltraNurse-physician discussion took place. This discussion specifically focused on the clinical contribution, but when there was a change in diagnosis, these would all be confirmed and double-checked by the treating physician. Changes within the ICU nursing scope of practice did not require immediate notification of a physician and could be discussed afterwards, whereas findings and accompanying diagnostic and management changes outside of the ICU nursing scope of practice were communicated to the physician during a visit for consultation. An immediate consultation took place within 10 minutes and was often initiated with a phone call, requesting the bedside presence of the treating physician.

The ICU nursing scope of practice was defined with the help of the nursing diagnosis, intervention, and outcome classifications (Carpenito, 2012; Mccloskey Dochterman et al., 2007; Moorhead et al., 2018). Management adjustments within the ICU nursing scope consisted of the following: change in fluid management (increasing/decreasing fluid administration, administration of pre-prescribed diuretics, and increasing ultrafiltration in continuous renal replacement therapy), vasoactive dose titration, or change in ventilatory settings. Other suggested management changes resulted in an immediate visit of the physician for consultation to discuss the diagnosis and treatment plan.

#### 2.3.4 Further data collection

The completed case report forms, along with other data collected from the electronic medical records, were stored within the Castor ‘Electronic Data Capture’ system. Execution of suggested changes of management within the first 8 hours were verified in the electronic medical record. The 8 hour interval was chosen because of the duration of a regular UltraNurse shift. Also, an UltraNurse or a colleague from the relieving shift would certainly encounter a physician during this interval to discuss the clinical contribution and (suggested) management/diagnosis changes.

The following patient characteristics were collected: age (years), sex (male/female), Body Mass Index (kg/m2), time until receiving UltraNurse thoracic ultrasound (in days), number of diagnoses, if mechanical ventilation was administered during ultrasound, Positive End Expiratory Pressure (in cmH2O), medical/surgical type of admission, reason for admission, and the medical history. The Sequential Organ Failure Assessment score and the arterial partial pressure of Oxygen/Fraction inhaled Oxygen ratio (in mmHg) were collected specifically to display severity of disease (Ferreira et al., 2001; Ranieri et al., 2012).

### 2.4 Data analysis

The sample size was calculated with an expected ability to initiate a change of management (or population proportion) after ultrasound of 35%, a margin of error of 10% with a Confidence Interval of 95% (95% CI). One patient could undergo multiple ultrasound examinations; when adjusting for 15% of data nesting within cases, a sample size of 101 examinations was deemed sufficient. This is comparable to similarly designed studies where sample sizes varied around 100 ultrasound examinations with similar abilities to initiate a change of management (Haji et al., 2018; Pontet et al., 2019). Data analysis was performed with IBM SPSS Statistics for Windows, Version 24.0. Armonk, NY: IBM Corp.

Patient characteristics were presented as frequencies when categorical (n) with a percentage (%), and when continuous either as mean with standard deviation (± SD) or median with Inter Quartile Range (IQR) when appropriate.

We analyzed primary and secondary outcomes within the group where the clinical contribution of the ultrasound was scored as leading to ‘a change of initial impression’ to exclude changes of management already anticipated by the clinician. Clinical contribution of the ultrasound was further presented as: ‘no contribution’, ‘confirmation’, or ‘change of initial impression’

Outcomes were specifically presented as frequencies (n) and percentage (%) of total cases. Per scope of practice, the 95% CI was added. The frequency with which an UltraNurse did perform ultrasound examinations was estimated by dividing the total examinations by cumulative worked shifts amongst UltraNurses.

### 2.5 Ethics approval and consent to participate

This study was a preplanned subanalysis of a study to investigate the impact of thoracic ultrasound on clinical decision-making and was approved by the local institutional review board (Netherlands Trial Registration NL9407, VUmc 2020.011, prospectively registered on: 13–11–2020). The requirement for consent was waived.

## 3 Results

Between February and August 2021, 102 ultrasound examinations were included for 65 ICU patients. Patient characteristics at time of examination are presented in Table 1. The median age was 68 years, and the majority were female. The mean Sequential Organ Failure Score was 9. COVID-19 pneumonia was the predominant reason for admission, and most ultrasounds were performed in mechanically-ventilated patients.

**Table 1:** Characteristics of included patients at time of UltraNurse thoracic ultrasound examination.

*Total patients (N* *=* *65)*	*N (%)*	*Median [IQR]*	*Mean ± SD*
Sex (female)	15 (23)		
Age (years)		68 [61 - 74]	
Time from admission to first ultrasound (days)		5 [1–13]	
Type of admission^a^			
Medical	50 (77)		
Surgical	14 (21)		
Other	1 (2)		
Severity of disease			
Mechanical ventilation during ultrasound	89 (87)		
PaO2/FiO2 (mmHg)		144 [121 - 187]	
PEEP (cmH20)			10 ± 3
SOFA			9 ± 3
BMI (kg/m2)			28 ± 7
Number of diagnoses			4 ± 2

Used abbreviations: BMI = Body Mass Index, No. = number, PEEP = Positive End Expiratory Pressure, PaO2/FiO2-ratio = Perfusion/Fraction inspired Oxygen-ratio, SOFA = Sequential Organ Failure Assessment.

a See supplement 4 for further specification of admission types and medical history.

### 3.1 Outcomes

In Table 2, the suggested management changes after ultrasound examination, and the percentage of changes executed within the first 8 hours are presented per scope of practice. Twenty-seven (26%) of the ultrasounds suggested a change of management. Almost all of these changes (96%) were executed within 8 hours. Of all management changes, those most often executed were within the nursing scope of practice (56%). Within the nursing scope specifically, 19 (44%) of ultrasound examinations led to a change in fluid management.

**Table 2:** UltraNurse thoracic ultrasound and its effects on outcomes studied.

	*N*	*%*	*95% CI*
Total UltraNurse ultrasound examinations^a^	102		
Suggested change of management per ultrasound	27	26	[18%−36%]
Change executed within 8 hours	26	96	
Change not executed	1	4	
Total specific management changes^b^	43		
Within ICU nursing scope of practice^c^	24	56	[41%−71%]
*Executed*			
Change in fluid management	19	44	
Negative fluid balance^d^	10	23	
Positive fluid balance^e^	9	21	
Change in ventilatory settings	4	*	
Vasoactive dose titration	1	2	
Outside ICU nursing scope of practice^f^	18	42	[27%−57%]
*Executed*			
Drainage of pleural effusion	4	9	
Additional Imaging	4	9	
Extubation	4	9	
Bronchoscopy	1	2	
Start inotropics	1	2	
Intubation	1	2	
Perform COVID-PCR test	1	2	
Rethoracotomy for pericardial drainage	1	2	
*Abstained*			
Extubation	1	2	
*Not executed*			
Fluid challenge	1	2	
Exclusion due to agitation or technical issues^g^	0		

Used abbreviations: PCR = Polymerase Chain Reaction, PEEP = Positive End Expiraroty Pressure.

a The results presented are from within the group where UltraNurse thoracic ultrasound was scored as leading to ‘a change of initial impression’.

b One thoracic ultrasound examination, in one patient, could lead to multiple management changes.

c Defined with help of the nursing diagnosis, intervention and outcome classifications (Carpenito, 2012; Mccloskey Dochterman et al., 2007; Moorhead et al., 2018).

d Management aimed at decreasing fluid balance, e.g. decreasing maintenance fluids or administering pre-prescribed diuretics, increasing ultrafiltration in Continuous Renal Replacement Therapy.

e Management aimed at increasing fluid balance, e.g. increasing maintenance fluids, administering a fluid challenge, decrease ultrafiltration in Continuous Renal Replacement Therapy.

f After (immediate) physician visit for consultation; UltraNurse thoracic ultrasound examinations were used in decision making.

g As stated on the case report form under particularities.

In Table 3, ultrasound findings are presented. In 97% of patients, pathologic findings were present. Most frequent findings were atelectasis (58%), pleural effusion (58%), pneumonia (51%) and pulmonary edema (51%).

**Table 3:** UltraNurse thoracic ultrasound findings.

	*N*	*%*
Pathological UltraNurse thoracic ultrasound findings per examination^a^	100 (97)	97
Atelectasis	58	58
Pleural effusion	58	58
Pneumonia	51	51
Pulmonary edema	51	51
Pneumothorax	4	4
Hypovolemia	11	11
Hypervolemia	3	3
Normovolemia	1	1
Pericardial effusion	1	1
Other	17	17

a One thoracic ultrasound examination, in one patient, could lead to multiple pathological findings.

In Table 4, formal changes in diagnoses are presented. The diagnoses changed after 7% of UltraNurse thoracic ultrasounds. In all cases, these diagnoses were confirmed by the treating physician with additional thoracic radiography, computed tomography, or a diagnostic puncture.

**Table 4:** Formal diagnosis change after ultrasound examination.

	*N*	*%*
Formal change in diagnosis	7	
Lung consolidation	1	14
Hypovolemia	1	14
(Suspected) COVID-19 pneumonia	1	14
Cardiac tamponade	1	14
Pleural effusion/empyema	1	14
Pneumothorax	1	14
Liver cyst suggested	1	14

The clinical contribution of UltraNurse ultrasound was scored as follows: 34 (33%) of the ultrasound examinations led to ‘a change in initial clinical impression’, 57 (56%) examinations ‘confirmed the initial impression’, and 11 (11%) examinations were scored as ‘not contributing’.

On average, an UltraNurse performed one thoracic ultrasound examination per four shifts (102 examinations/409 worked shifts).

## 4 Discussion

The main findings of this prospective observational study on the effect of UltraNurse thoracic ultrasound on management of the adult ICU patient were as follows: 1. UltraNurse ultrasound led to a change in management in around one-fourth of cases, of which 96% are executed within the first 8 hours; 2. Ultrasound examinations commonly detected pathologic findings (97% of cases), of which atelectasis (58%), pleural effusion (58%), and pulmonary edema (51%) were most common; 3. Formal diagnosis change was made in 7% of cases, of which some were life-threatening and needed immediate management; 4. On average, one ultrasound examination was performed per four shifts.

Although UltraNurse ultrasound examinations led to a smaller percentage of management changes than physician thoracic ultrasound (26% vs. 42%), this is still a substantial percentage of patients (Heldeweg et al., 2022). Additionally, almost all (96%) of the management changes suggested by the UltraNurse were executed within the following 8 hours, indicating that UltraNurse ultrasound led to management changes that were highly executable. This high execution rate was probably a result of the constant bedside presence of the UltraNurse.

The difference in percentage of formal diagnosis change after UltraNurse ultrasound (7%) vs. physician ultrasound (44%) was even larger (Heldeweg et al., 2022). For both the difference in management and diagnosis changes, there are multiple possible reasons. UltraNurse ultrasound has less effect on changes than physician ultrasound probably because of the more extensive ultrasound skill set with extended scope of practice for physicians. Also, UltraNurse ultrasound is more often used as a tool for monitoring of therapy, whereas physician ultrasound is often used to diagnose new problems in the early clinical stage. In this stage, there probably was a larger clinical uncertainty and thus increased detection of pathology (Heldeweg et al., 2022). The percentage of diagnosis change seems low for UltraNurse ultrasound, but this was still a considerable amount since a new diagnosis often warranted immediate consultation of a physician for sometimes life-saving treatment.

Another important, although not surprising, finding is that in our study, most of the changes in management were within the nursing scope of practice and related to fluid management and ventilator settings. This has some important implications for all scopes of practice. First, UltraNurses used ultrasound to help guide practice within their scope, possibly enhancing nursing outcomes (Carpenito, 2012; Mccloskey Dochterman et al., 2007; Moorhead et al., 2018); Second, UltraNurse ultrasound mainly helped in fluid management optimization —which is a notoriously difficult goal within ICU practice— but important because a positive fluid balance is associated with increased morbidity and mortality (Marik et al., 2020; Marik, 2014; Melenovsky et al., 2015; Banerjee et al., 2007); Third, ultrasound also initiated changes of ventilatory settings in 10% of cases. Changes in ventilatory settings are already an important part of the European ICU nursing scope because the ICU nurse is constantly at the bedside and therefore best suited to monitor progress (Carpenito, 2012; Mccloskey Dochterman et al., 2007; Moorhead et al., 2018). Thus, UltraNurse ultrasound might also support ventilatory management.

Outside of the nursing scope of practice, there are also some important pathologic findings detected by UltraNurse ultrasound. First, it can initiate (immediate) consultation of the physician for life-saving treatment; for example, drainage of a cardiac tamponade, large pneumothoraces, or pleural effusions. When the UltraNurse stated the new suggested diagnosis when contacting the treating physician, the need for immediate bedside presence was clear, and this likely limited the interval from diagnosis to treatment. In all cases, the new diagnosis was confirmed by the physician.

Although ultrasound may be an important tool to manage ICU patients, UltraNurse ultrasound was performed just once per four shifts. Reasons for this could be: the study period where nursing workload increased immensely during the COVID-19 pandemic, and general nursing responsibilities did not always allow UltraNurse ultrasound to fit into daily practice.

There are also theoretical benefits of UltraNurse ultrasound for the individual ICU nurse. Pathophysiological knowledge is increased by ultrasound training and expands the scope of practice of the ICU nurse; this helps support decision-making and increases the UltraNurses’ confidence in interdisciplinary discussion. This makes the UltraNurse a more impactful and equal ICU team member. In addition, ultrasound education can be seen as a new and motivating challenge. This all could possibly increase job satisfaction.

Strengths of this study include its novelty, achieved sample size, and straightforward observational design. The simple data collection procedures, together with the usage of readily available data from the electronic medical record, minimized information bias and missing values.

### 4.1 Limitations

This study also has several limitations. Selection bias could have been introduced because patients were selected based upon an indication for ultrasound; these patients may already be more likely in need of a diagnosis/management change. Some reporting bias cannot be excluded because the role of the physician in deciding on management changes was not recorded and could potentially have influenced results. However, to address these two forms of bias, the clinical significance (contribution) was scored collectively after physician-UltraNurse discussion took place to minimize these influences. Lastly, half of the included ICU patients were admitted because of respiratory failure due to COVID-19, possibly increasing pathological lung ultrasound findings, since these are more prevalent and distinct in COVID-19 pneumonia (Heldeweg et al., 2021; Haaksma et al., 2020). However, applying UltraNurse ultrasound in these patients was still helpful in guiding management.

Future research should focus on causal designs and patient related outcomes to further substantiate the effects of UltraNurse thoracic ultrasound. Our relatively low frequency of ultrasounds performed per shift, together with the novelty of the UltraNurse, possibly indicate that fit within daily workflow could be further optimized, and therefore an implementation study is also warranted.

## 5 Conclusions

In critically ill adult ICU patients, UltraNurse thoracic ultrasound led to a change of management in more than a quarter of cases, of which almost all management changes were executed within the following 8 hours. In a small subset of patients, critical new diagnoses were detected with crucial management consequences. These promising outcomes justify larger validation studies of UltraNurse thoracic ultrasound.

## Funding sources

Funding y by the Department of Intensive Care Medicine, Amsterdam University Medical Centers, location VUmc, Amsterdam, The Netherlands.

## What is already known

• Thoracic ultrasound is a valuable tool in helping physicians with clinical decision-making and management of adult intensive care unit patients
• Intensive care unit nurses trained in thoracic ultrasound, so-called UltraNurses, demonstrated rapid learning trajectories, but their effects remain uninvestigated (Morreale Tulleken et al., 2019)

## What this paper adds

• UltraNurse thoracic ultrasound led to a change of management in more than a quarter of cases
• Almost all changes were executed within 8 hours after ultrasound examination and were predominantly within the intensive care nursing scope of practice
• Sometimes these management changes had life-saving potential

## Data availability

Datasets used during this study are available on reasonable request by contacting the corresponding author.

## CRediT authorship contribution statement

**Thomas Smits:** Methodology, Formal analysis, Data curation, Writing – original draft, Writing – review & editing. **Micah Heldeweg:** Methodology, Formal analysis, Data curation, Writing – original draft, Writing – review & editing. **Amy Morreale Tulleken:** Writing – original draft, Writing – review & editing. **Brian Verlaan:** Data curation, Writing – review & editing. **Lonneke Floor:** Data curation, Writing – review & editing. **Alwin Eijsenga:** Writing – review & editing. **Erik Lust:** Writing – review & editing. **Harry Gelissen:** Writing – review & editing. **Armand Girbes:** Writing – review & editing. **Paul Elbers:** Writing – review & editing. **Pieter Roel Tuinman:** Methodology, Formal analysis, Data curation, Writing – original draft, Writing – review & editing.

## Declaration of Competing Interest

None declared in authorship and conflict of interest statement.

## Appendix Supplementary materials

Image, application 1

Image, application 2

Image, application 3

Image, application 4
