# Supplementary material for: Effects of nurse delivered thoracic ultrasound on management of adult intensive care unit patients: A prospective observational study
Source: Int J Nurs Stud Adv. 2023 May 29;5:100135. doi: 10.1016/j.ijnsa.2023.100135 (PMC11080432; doi:10.1016/j.ijnsa.2023.100135)
Supplement: Supplementary file 1 [file mmc1.docx]

**Supplement 1**

*Fig 1. Flow chart of study procedures*

**
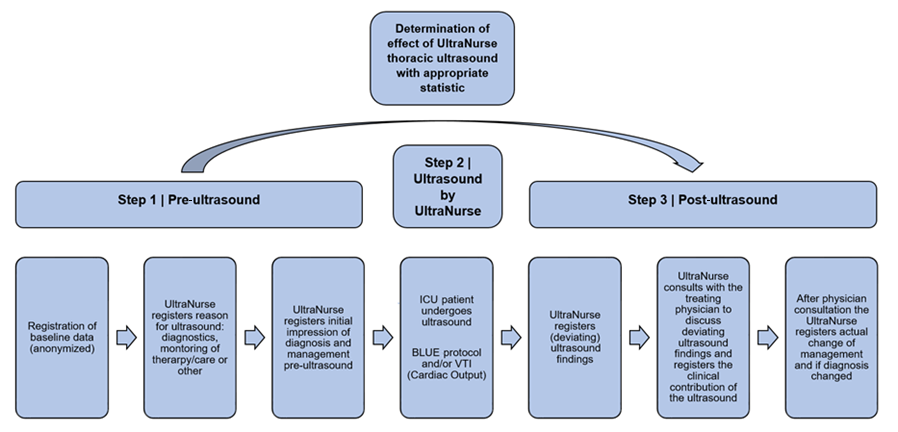
***Used abbreviations: BLUE = Bedside Lung Ultrasound in Emergency, ICU = Intensive Care Unit, VTI = Velocity Time Integral.*
