# Supplementary figures and images for: Effects of nurse delivered thoracic ultrasound on management of adult intensive care unit patients: A prospective observational study
Source: Int J Nurs Stud Adv. 2023 May 29;5:100135. doi: 10.1016/j.ijnsa.2023.100135 (PMC11080432; doi:10.1016/j.ijnsa.2023.100135)

**Supplement 2**

*Fig 2. Case report form*


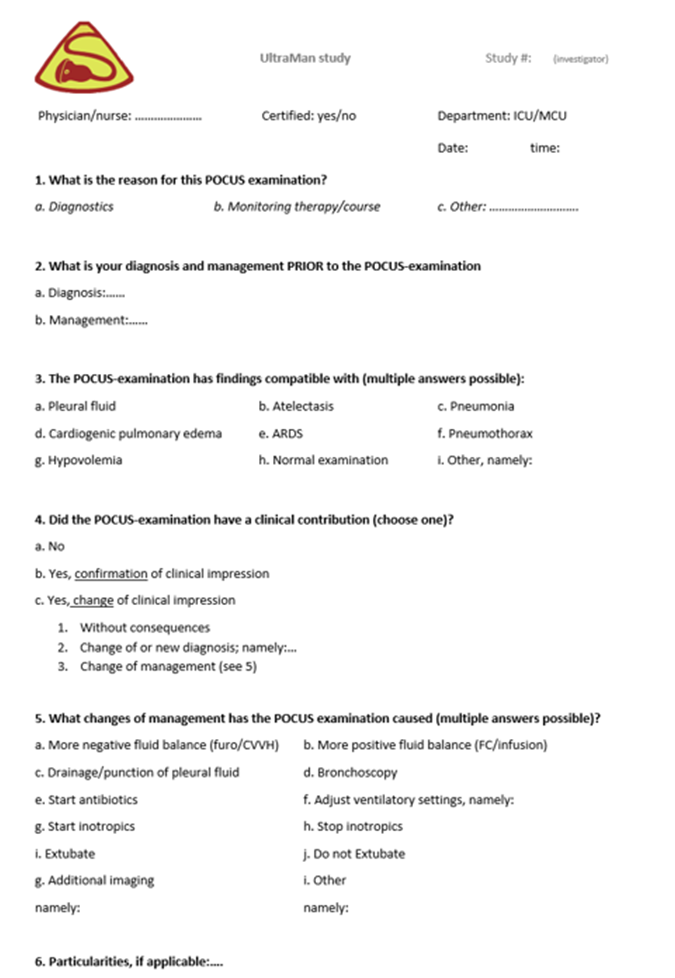

Supplement: Supplementary file 2 [file mmc2.docx]
