# Supplementary material for: Effects of nurse delivered thoracic ultrasound on management of adult intensive care unit patients: A prospective observational study
Source: Int J Nurs Stud Adv. 2023 May 29;5:100135. doi: 10.1016/j.ijnsa.2023.100135 (PMC11080432; doi:10.1016/j.ijnsa.2023.100135)
Supplement: Supplementary file 3 [file mmc3.docx]

**Supplement 3**

*Fig 3. The UltraNurse thoracic ultrasound examination (published with permission) [11]*


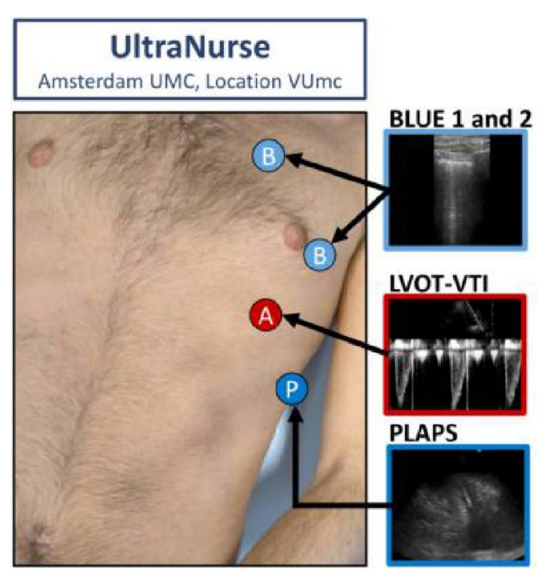


**Left hemithorax marked with the Bedside in Lung Ultrasound in Emergency points (B- and P-markers) and Velocity Time Integral probe placement (A-marker) as used in the UltraNurse ultrasound examinations. Note that de B- and P-markers are scanned bilaterally.*

*Used abbreviations: BLUE = Bedside Lung Ultrasound in Emergency, LVOT-VTI = Left Ventricular Outflow Tract – Velocity Time Integral, PLAPS = Posterolateral Alveolar and/or Pleural Syndrome*

**The UltraNurse training program**

The UltraNurse training program consisted of the following phases and key points:

- Theoretical education phase - one day
  - Learning how to use the basic functions of SonoSite Edge II (ultrasound machine)
    - Base functions: depth, gain, exam profiles and administration of patient characteristics
    - Probe types and differences (linear and phased-array)
    - General troubleshooting
  - Education in common pathology and cardiac output measurement (see figure 3):
    - Based on the Bedside Lung Ultrasound in Emergency protocol [8, 9]
    - Velocity Time Integral for cardiac output estimation [10]
    - Registration of findings in the standardized format
    - Bedside demonstration on probe placement by an ultrasound certified physician
- Hands-on clinical phase - around 3 months
  - The first five ultrasounds were directly supervised by ultrasound certified physicians or ICU nurses
  - Then every fifth ultrasound was directly supervised and scored in a standardized manner to measure progress
  - When the UltraNurse-trainee reached >90% scores when supervised, the examination phase could be started
    - UltraNurse-trainees would reach certification if they had a supervised score of 100% for five consecutive ultrasounds
  - Most UltraNurse-trainees reach proficiency in 23 weeks [11]
