# Supplementary material for: Effects of nurse delivered thoracic ultrasound on management of adult intensive care unit patients: A prospective observational study
Source: Int J Nurs Stud Adv. 2023 May 29;5:100135. doi: 10.1016/j.ijnsa.2023.100135 (PMC11080432; doi:10.1016/j.ijnsa.2023.100135)
Supplement: Supplementary file 4 [file mmc4.docx]

**Supplement 4**

*Table 5: Further specification of admission types and medical history*

| **Reason for admission** | *N* | *%* |
| --- | --- | --- |
| Respiratory failure |  |  |
| Covid-19 pneumonia | 30 | 46 |
| Pneumonia | 2 | 3 |
| Empyema | 1 | 2 |
| Pulmonary embolism | 1 | 2 |
| COPD | 1 | 2 |
| Threatened airway with subcutaneous emphysema | 1 | 2 |
| Cardiovascular |  |  |
| Cardiac arrest | 6 | 9 |
| Acute heart failure | 1 | 2 |
| Medical |  |  |
| Distributive shock; septic | 1 | 2 |
| Hypovolemic shock; dehydrated | 1 | 2 |
| Renal failure; hyperkalemia | 1 | 2 |
| Surgical |  |  |
| Upper gastrointestinal tract | 9 | 14 |
| (Multi-)trauma | 4 | 6 |
| Heart valve | 1 | 2 |
| Aortic/Aneurysm | 2 | 3 |
| Neurological |  |  |
| Viral infection | 1 | 2 |
| Epilepsy | 2 | 3 |
| **Medical history** |  |  |
| Cardiovascular | 41 | 33 |
| Pulmonary | 21 | 17 |
| Gastrointestinal | 8 | 6 |
| Renal | 3 | 2 |
| Neurologic | 4 | 3 |
| Psychiatric | 3 | 2 |
| Other | 45 | 36 |

*Used abbreviations: COPD = Chronic Obstructive Pulmonary Disease*
